# The need for social network analysis for the investigation of affective variables in second language acquisition

**DOI:** 10.3389/fpsyg.2022.983698

**Published:** 2022-08-08

**Authors:** Mengzhong Wang

**Affiliations:** ^1^School of Foreign Languages, Hunan University of Arts and Science, Changde, China; ^2^Lyceum of the Philippines University–Batangas, Batangas, Philippines

**Keywords:** affective variables, complex dynamic systems theory (CDST), second language acquisition (SLA), social network analysis (SNA), investigation

## Abstract

Considering the inherent developmental nature of language learners’ affective variables (e.g., their motivation, grit, foreign language enjoyment, and boredom), nuances of the development of these constructs need to be approached from a complex dynamic systems theory (CDST) perspective. Among the qualitative research methodologies compatible with the CDST is the social network analysis (SNA) with the interconnectedness and interdependence of systems within a social network at its core. In this article, an overall introduction to SNA is presented first and then followed by a review of the limited existing literature on second language acquisition (SLA) studies. Then, I argue why this innovative research method is suitable to investigate the dynamic nature of L2 learners’ affective variables in the social network of classroom learning. I also suggest several relevant research questions that can potentially be formulated and answered using the SNA. The article ends with conclusive remarks on the need for a more extensive use of innovative CDST-compatible research methods such as SNA in the prospective SLA line of research.

## Introduction

From the mid-20th century onward, human and social sciences have made great contributions to the theorization of complexity, and they have made use of the considerable insights it has provided (Capra and Luisi, 2014). Social sciences have shown an ever-growing interest in humans, their behavior, and interactions as the main purposes of research. The social world has been interpreted differently across intellectual schools. For instance, in humanistic psychology, the social interaction between individuals takes place in a nurturing environment where their emotions are fully acknowledged via rapport and emotional scaffolding (refer to Buhler, 1971). On the other hand, the constructivist school of psychology puts emphasis on the mediating role of individuals in their social interaction in which cognitive development is realized out of the mediation of more experienced individuals (refer to Prawat, 1999). Still, embracing human behavior and relations in their totality is not an area that social scientists have easily lost sight of Byrne (2011). A discourse influenced by the complexity theory, with key inherent terms such as interconnectedness, dynamicity, interactions, and network, in present-day social and human sciences is followed back to Smith’s (1723–1790) notion of the invisible hand, which refers to the unexpected adverse effects of certain social measures, or the descriptions of the politics of the time by Burke (1729–1797). However, new ways of tracing the complexity line of thinking and research on social sphere in the past two centuries seem to have much to contribute to social sciences, communication studies, and applied linguistics.

As interconnectedness lies at the core of complexity dynamic systems theory (CDST) research, discovering which factors (individuals, groups, or any other actor) form networks and contribute to the emergence of complex constructs is a matter of inquiry. This means that how the associations of systemic actors make up an integrated totality in the social network of a given environment seems to be a rich domain for CDST investigations based on social network analytic (SNA) procedures (Hiver and Al-Hoorie, 2019). Within the domain of L2 affective variables, the literature on some positive emotions such as enjoyment has highlighted the susceptibility of these emotions to the social atmosphere of the classroom and the behavior and feeling of teachers in the class. For instance, this social aspect has been reflected in almost all scales developed for measurement of foreign language enjoyment. Thus, it is postulated that the interaction of agents in the social interaction of the environment of the classroom should be the focus of L2 researchers by application of suitable methods for this purpose. One of the most suitable methods to explore the emergence of positive emotions out of the social network of interactions in the ecology of an L2 classroom is SNA. One noticeable feature of recent developments in research on L2 affective variables is the expansion of the nomological networks of these variables. However, there is a difference between the nomological network and the social network of these variables. The former reflects the network of related constructs with the L2 variable of interest such as boredom or enjoyment, and the latter represents the social structure underpinning the emergence of a given L2 affective variable. In the following, I introduce SNA first as a CDST compatible research approach and then justify why and how it can be employed as a primarily qualitative approach to research in second language acquisition (SLA) and more specifically in exploring affective variables. I will cite exemplary research studies that used this analytical method and discuss the pedagogical implications of this approach in SLA research. Suggestions are also made for further investigations on affective variables in SLA using this qualitative approach for research.

## Social network analysis and complex dynamic systems theory

In SNA, a network is described as a center with multiple nodes comprised of intertwined systems (representing individuals, groups, organizations, etc.), their interactions, and underlying mechanisms that, as a whole, comprise a basic set of the surrounding-complicated social sphere (Knoke and Yang, 2008). Researchers seeking to explore complex and dynamic systems such as language learners, learner emotions, and personality factors find this definition helpful as whatever system they hope to investigate is only seen as one out of multiple nodes integrated inside this network marked by many interconnections.

In the early to mid-1900s, SNA was born out of social anthropology and sociology, with a focus on mapping social structures through innovative descriptors such as fabric, web-like, or inter-woven relationships (Prell, 2012). Gradually, until the 1970s, such metaphors got established in the social network theory, and more specialized approaches and technical uses appeared out of the publications by Parsons (1951) as well as Parsons et al. (1953). Since then, this social network analysis (SNA) has become so widespread that there are formal procedures firmly established for collecting, analyzing, and applying the data obtained from the network of interest (e.g., an L2 classroom learning context). The basis of the full range of SNA is the fundamentally presumed significance of inter-links among interwoven systems or units that are continually interacting with each other (Scott and Carrington, 2011; Scott, 2017). That is why relational data are essential in SNA to map models to represent relation-based constructs, emotions, behaviors, or procedures (Yang et al., 2017). As a clear instance of what is implied by relational data, I can think of the network of language learners in a class. Each student can be considered a system within a network with connections to others (peers or teacher) in the entire language learning context. The association of two systems is a feature of a dyad or a pair (as a more familiar term used in classroom learning), a summative value, here for two students (language learners), not only one or the other student. These kinds of connections can include language interactions, behavioral effects, emotion expressions, affiliation with the same group, course content exchange, sharing of knowledge, or personal appraisal of each other (Borgatti et al., 2018), which could possibly be all directly investigated with(out) reliance on individual characteristics of included systems.

Social network analysis contrasts with traditional approaches to research as the latter primarily tend to conduct a variable analysis using attribute data. They may put forth research questions addressing students’ commonalities or points of departure with the help of non-relational data. This is different from what SNA researchers hope to gain from analyzing social networks. Thus, research designs are different, and so are the sample selection method, measurements made, and use of the findings of data. There are certain basic assumptions underlying social network patterns and research approaches, as summarized here (Wasserman and Faust, 1994; Kadushin, 2012; Prell, 2012).

First, it is assumed that individual systems and their behavior or affective qualities within a network are not independent of each other. Rather, they are connected with and, thus, dependent on each other. Second, systems exchange resources (including knowledge and effects) through associations of individual systems that are, in fact, reciprocal pathways and try to keep up this dependence on each other. Third, the network structure is marked by inherent stability in related pathways. Mind that this stability does not mean being fixed or static; rather, it means that the pathways strike a balance with each other. Fourth, certain limits (or in contrast chances) are created for the functioning of every single system within the network. Finally, the whole relational structure of the network receives feedback from the functioning of every single constituent system.

For information representation of systems (e.g., language learners) and mutual interactions within the network (e.g., a learning class), visual matrices, concept maps, or sociograms are used in SNA. This is somehow similar to concept mapping, but there are certain complicated computational analyses added in a number of SNA analysis packages to rely on graph theory. Another distinctive feature of SNA from concept mapping is that the former has a heavier emphasis on the structure analysis and modeling of networks in which the systems lie as well as the relational orders and effects of the interdependent network on the constituent systems (Mercer, 2015). However, sometimes, in later phases of analysis, quantitative procedures are also needed in a number of SNA analytical devices, but still the qualitative type is usually dominant.

At the heart of SNA lies the quality of interconnectedness, which means every system is connected and somehow associated with all other systems within the more inclusive context (Scott, 2017). For example, in an L2 class, every student (language learner) can be conceived as related to other language learners (i.e., peers). According to Carolan (2014), systems can be conceived as networked when linked to each other in a phenomenological, authentic, or practically significant fashion. Interconnection is considered a major feature in CDST investigations; thus, discovering networked systems and untying how their connections comprise an integrated whole might be a fruitful area for CDST-based investigations by SNA. Although the main focus of SNA is to visualize the systemic network structure and their relational architecture, several layers of analysis can be conducted. SNA also seeks to see what the contribution of this higher-order structure is to the unraveling of the behavior or affective qualities of systems subsumed under it (Carolan, 2014). Significant properties characterize these two layers, which may delve into significant structures or mechanisms. SNA researchers call it the dual individual or structure (Hanneman and Riddle, 2005, 90). With this duality feature, systemic networks can help to explore interconnection patterns and illuminate the overt or covert issues on the system scale and map the dynamic relations.

It is noteworthy that in SNA, the analysis unit is not merely the person or a local system and, instead, an exhaustive group made up of a company of persons or systems and the associations among them (Prell, 2012; Yang et al., 2017). This information is developed in SNA as represented by points and paths. The former (which are termed as nodes or actors) stand for persons or systems and their objectives, perceptions, emotions or actions. The latter (in SNA termed as ties and edges) that go from one point to another and link them with each other indicate the correlational and causal interrelationships among them.

With all this in mind, I feel that SNA is an appropriate approach to studies on language learning and development of learner-related factors including affective variables. I will now turn to a brief look at how SNA has been applied so far in SLA studies. Exemplary research studies will be reviewed. Then, we will move on to see what research question types can be formulated in an SNA research design in SLA studies to explore the dynamic development of affective variables.

## Social network analysis in L2 studies

It is fortunate that SNA is a CDST-appropriate research methodology that has shown to be efficient, yet not prevalent, in the history of SLA research. A good instance is a pioneering attempt to apply SNA to the language acquisition phenomenon published in a special issue of the International Journal of the Sociology of Language (de Bot and Stoessel, 2002; Wiklund, 2002), the processing of language (Hulsen et al., 2002), the maintenance and retrieval of language (Raschka et al., 2002; Stoessel, 2002) and competencies and performance of language (Smith, 2002). The newest use of SNA in applied linguistics has been the application of more formal SNA methods, i.e., in published research studies on how communication is perceived and shown in practice (Gallagher and Robins, 2015; Gallagher, 2019).

Recent SNA research has shown interest in the process of network development through the passage of time, and has used simulating techniques to investigate existing problems related to network dynamics. Currently known as dynamic network analysis, this newly developed research domain employs complicated mathematical maps to delve into networks that are characterized as adaptive and dependent on time. In the light of new dynamic network analysis procedures, it is possible to see how they have been used in domains related to applied linguistics and to see how their use can be extended to the exploration of issues in SLA. Moreover, the considerable progress and the emergence of big data along with more robust or automatized analytic procedures make it possible to develop and test models of full-network data (also known as meta – and high-dimensional networks) having many nodes and sophisticated associations within the network.

An example of SNA applied in the SLA domain is the study designed by Gallagher and Robins (2015) to explore the details of constructing communication networks in English as a second language (ESL) class. They also sought to find how these interconnected bonds influence students’ willingness to communicate (WTC), an important learner-related personality trait, in an English for academic purposes (EAP) course (Gallagher, 2019). EFL class learning is a context ripe for formal or informal situations that require language learners to communicate with pairs or take part in group-based tasks or have presentations in class. Thus, the researchers aimed to see to whom ESL learners opened their heart and shared their personal issues. This relational information was investigated to compare the self-organization of networks among students affiliated with a common linguistic and cultural group and students who have intercultural social bonds.

This research hypothesized that students’ WTC in the second language was socially shared as a function of the relative positions of individuals in a social network and diverged significantly across interactions in small and big groups. To this aim, a sample of 75 EAP language learners were selected from a tentative, but not credit holding EAP program at an English college, who argued that the EAP program comprises a relatively well-established system of individual members interacting with each other in the pursuit of a shared goal. The students used open recalls to mention the names of about 10 peers with whom they had shared important matters in the past 14 days (Gallagher and Robins, 2015, p. 942). Intracultural nodes were specified to code the relational data, as the research participants and their peer candidates both shared a similar culture. Intercultural nodes were specified as the peer candidate being from a different culture from the main research participant. The language learner participants’ WTC was also measured using a revised edition of the WTC scale. The students were supposed to self-rate the proportion of time they might want to spend communicating in second language in different social contexts on this questionnaire.

Gallagher and Robins used the XPNet analytic software and specified the model with two types of network tie at the same time so they could explore the intracultural nodes in comparison to the intercultural ones. They also aimed to examine the directed paths, in which the students choose or send nodes to other students directionally. Next, they determined the whole effect of the network such as how the baseline node, star, triangular, and bi-path effects could appear in the target dataset. These researchers went further to examine the interaction effects of the network nodes and WTC in a second language (e.g., sender or receiver effects, negative difference, or positive product effects) with the aim of testing the hypotheses of the research. Finally, the analyses led to the anticipation of WTC in the second language being generally correlated with more intense network functioning, large-group second language WTC being correlated with a greater level of the network, and small group second language WTC being common among network nodes.

These researchers concluded that group structure was largely coordinated across intracultural nodes. However, the intercultural nodes did not seem to present with similar self-organizing qualities. The findings of this research contained many details about intra-network structure and relational nodes (Gallagher and Robins, 2015). They also showed that students having a lot in common as concerns the second language WTC in one-on-one or small-group situations shared specific intracultural social bonds in the network. Also, second language WTC in large-group and presentation contexts was spread based on popularity/level despite the fact that it was restricted to students’ intracultural nodes. However, this research provided no support to the idea that language learners whose WTC is high were more effective in their network nodes in any opportunity for communication.

## Social network analysis and a complex dynamic systems theory approach to study L2 learners’ affective variables

Following the dynamic turn in the psychology of language learning (refer to Dörnyei and Ryan, 2015), some L2 affective variables have been revealed as complex dynamic systems. One key property of complex systems is that they undergo change under the influence of external factors and interaction with the environment. However, this property of complex systems reflected in the emergence of L2 affective variables has not been explored by using suitable CDST-compatible methods. Most of the methods used in support of the complex nature of some emotions such as anxiety, boredom, and enjoyment in the field of SLA have been mainly bound to the self-organization of these emotions under the influence of internal factors (self-organization). Therefore, SNA originated from the social psychology of language learning and can cater to the needs of L2 researchers to explore how social factors contribute to the emergence and change in L2 affective variables. The existing body of research on SLA so far has shed light on multiple affective variables that can be involved in foreign or second language learning. Examples are learners’ self-confidence (de Saint Léger and Storch, 2009; Peng and Woodrow, 2010; Lee and Lee, 2019), L2 anxiety (Macintyre and Legatto, 2011; Lee, 2019; Lee and Lee, 2019), burnout (Derakhshan et al., 2022b), and different types of language learning motivation (MacIntyre et al., 2002; Yu, 2011; Khajavy et al., 2016; Lee, 2019; Lee and Lee, 2019; Pishghadam et al., 2021), rapport (Derakhshan et al., 2022a), and grit (Duckworth et al., 2007; Lee and Lee, 2019). Although these affective variables have long been in the interest of SLA researchers, studies enlightened by the CDST are still limited. As described by Dewaele and Li (2020), SLA has entered the third phase (i.e., the dynamic phase) of investigating affective variables (Wang et al., 2021), following the general and domain-specific phrases. This dynamic turn is marked by a concern for both positive affective variables (e.g., motivation, self-confidence, grit, and foreign language enjoyment) and negative constructs (e.g., demotivation, anxiety, and boredom). There has been a shift in SLA research to examine the development of learner-related affective variables by tracing their complex dynamic interactions.

The past few years have witnessed more CDST-compatible research designs in exploring L2 learners’ affective variables. Examples are the foreign language enjoyment literature (e.g., Elahi Shirvan and Taherian, 2018, 2020a,b; Elahi Shirvan and Talebzadeh, 2018a,b; Elahi Shirvan et al., 2020) and foreign language learning boredom literature (e.g., Elahi Shirvan et al., 2021; Kruk et al., 2021; Yazdanmehr et al., 2021). Among the innovative qualitative methods have been process tracing and ecological assessment. SNA has been employed to a less degree, as already reviewed in the previous section.

There are reasons why I think SNA should effectively be used to explore language learners’ affective variables. The recent literature on L2 learners’ affective variables in the light of the CDST revealed that all learners’ emotions, either positive or negative, are dynamically formed within the network of class learning, in which a number of actors are involved, the most important of which being the peers and the teacher. Moreover, as contended by Joe et al. (2017), in a safe, positive, and highly caring classroom context, mutual respect, peer interactions, and social connections among peers are best encouraged. In such a classroom environment, learning is combined with joy and strengthened interpersonal relations (Dewaele and MacIntyre, 2014). It should be acknowledged that teachers play a major role in creating such a caring class environment. The weight of teachers and the social air of an L2 classroom have been acknowledged to account for the emergence of some positive emotions like foreign language enjoyment. That is, unlike some negative emotions like foreign language classroom anxiety, L2 learners’ experiences of enjoyment are highly dependent on their teachers’ act and feeling in the class. More specifically, the contagious feature of positive emotions under the influence of L2 teachers’ emotional transfer in the social environment of an L2 class has been supported by some recent research (Talebzadeh et al., 2020; Shao and Parkinson, 2021). This environment in which language learners feeling more confident can experience increasing enjoyment and decreasing boredom is where students can feel safe to express their opinions and develop a friendly relationship and a social connection with their classmates. This might lead to an increase in EFL learner’s confidence and sense of cooperation, which in turn might increase the level of self-confidence, motivation, enjoyment, and decrease boredom in the classroom, as evidenced by a number of studies (e.g., Dewaele and MacIntyre, 2014; Khajavy et al., 2018; Dewaele et al., 2019; Elahi Shirvan and Taherian, 2021). More specifically, the contagious feature of positive emotions under the influence of L2 teachers’ emotional transfer in the social environment of an L2 class has been supported by some recent research studies (Talebzadeh et al., 2020; Shao and Parkinson, 2021). Therefore, the need for an appropriate method that can enable researchers to explore the potentials and affordances of L2 teachers with the social psychological accounts of both teachers and peers in an L2 class, in the emergence of L2 affective constructs, is felt more than before. It is suggested that SNA has the methodological capacity for this aim. SNA can hopefully consider the network of interacting forces in forming and growing language learners’ affective variables, and can effectively be used to model the social network of language learners’ growing emotions.

As SNA focuses on both the structural network that is born out of the relations and the effects the structural network can have on the behavior of single cases or groups (Carolan, 2014), it is one of the few analytical procedures that can be also employed to seek answers to a wide range of research questions (Kadushin, 2012). Given the exploratory and qualitative nature of SNA, the social network underlying the emergence of L2 affective variables are not assumed *a priori*. One can think of the following questions to guide a line of L2 learner affective variables in empirical studies on a descriptive scale with the aim of developing and validating formal models representing a network:

- To which systems or actors (e.g., teacher, peers, materials, and immediate classroom setting) is L2 learners’ affective construct (e.g., enjoyment, boredom, motivation, grit, etc.) related in a network of classroom learning?
- Which systems or actors are more effective and contributive and which ones are less so in a network feeding into the development of a certain affective construct in L2 learners?
- How is the fabric of the associations among these systems (e.g., teachers, learners, and peers) within the network?
- In this network of relations, what is the distribution of effects? How are the existing resources and materials shared? How is information shared among the systems? What pattern(s) do the collaborative acts and interactions form or follow?
- What structural patterns do the paths among the systems or actors within the network follow? How are these patterns characterized?
- For a certain desired (learning or else) outcome, at any level, how do the interactive systems affect the achievement of this outcome?
- How does the structure of a certain affective variable or more than one variable change or remain stable over time in the social network of classroom learning?

Going beyond the descriptive level, if one aims to inferentially interpret the use and effects of network models, questions that may guide fruitful lines of research can be:

- How do social networks that generate a certain affective variable develop and how do they exert higher-order self-organized effects?
- How can social networks host the emergence of, or change in, a certain L2 learner-related affective variable and how can the purpose or the network change?
- How do the constituent system elements of the social network of an L2 learning adapt and change through the passage of time?
- How can some sort of change to the relations (paths) among the systems appear within the network?
- How does the general development happen in the L2 learning social network? How do the part-by-part sequences of collaboration and interaction in the dynamic network of classroom learning affect the dynamic processes involved within the network?
- How do the sequences of communication and interaction in dynamic networks affect dynamic procedures in the network?
- What are the implications of the networks of two or more affective variables growing together with similar architectural structures yet with main differences in the final processes of network functioning?
- How can temporal changes that influence the performance and rigor of a network be traced and anticipated?

The list of potential research questions is not by any means exhaustive. More questions that are related to the interconnectedness of multiple L2- learner-related affective variables and others can be explored as efficiently using the SBNA method (Hiver and Al-Hoorie, 2019). Since visualization contributes to the facilitation of qualitative network data, several SNA-friendly tools have been already developed (e.g., NetMiner, ALlegroGraph, and NetDraw) that can help L2 researchers to suitably interpret network data. Thus, for the sake of better visualization of analyzed network data, it is suggested that these programs and tools be applied.

## Conclusion

The essentiality of exploring language learning, to which L2 learners’ affective variables is only a constituent part, from a dynamic approach, taking into account the variability in intra- and inter-individual performances and the need to study the processes of change in the reality of classroom learning, was first raised by. Later on, attention was further drawn to interdisciplinary and openness to external influences among the main properties of applied linguistics, which makes CDST a perfect match for studies in this domain. A complete overview of CDST in applied linguistics was then published by, describing the self-organizing, interconnected and co-adaptive nature of language learning and viewing language process as always emerging out of interactions. In order to systematically study the processes of change in the development of language learner-related variables including their emotions and bring firm evidence for that, effective methodological processes are needed. As suggested by Hiver and Al-Hoorie (2019), researchers of applied linguistics are expected to go beyond describing and theorizing changes in dynamic language systems and should conduct research to produce evidence for language development in the light of the CDST. Among several CDST-compatible qualitative research methodologies, SNA seems to be a perfect match for exploring language learners’ affective variables, most of which have scarcely been investigated dynamically before.

Although the SNA method is still less applied in research, in this study, I brought reasons why SNA is capable of revealing nuances of change in the development of language learners’ affective variables such as their motivation, grit, language learning enjoyment, and boredom. Some actual exemplary studies were reviewed, and a number of research questions that could be addressed by SNA were also formulated. Regarding the pedagogical implications of the possible findings of studies on L2 affective variables by SNA, it should be pointed out that the use of SNA sets the stage for a direct link between the methodological affordances of the method and teachers’ need to be aware of the social affordances of the environment of their classes. Thus, it is conjectured that the application of the SNA method can raise L2 teachers’ awareness of the influence spheres of social agents in the environment of the classroom. There are hopes that the future line of research can effectively use this innovative qualitative research methodology to reveal more about the nuanced development of L2 learners’ affective constructs embedded within the live social network of classroom learning.

## Author contributions

The author confirms being the sole contributor of this work and has approved it for publication.
